# Detection and Colonization of Multidrug Resistant Organisms in a Regional Teaching Hospital of Taiwan

**DOI:** 10.3390/ijerph16071104

**Published:** 2019-03-28

**Authors:** Yi-Ping Chen, Ching-Chao Liang, Renin Chang, Chen-Min Kuo, Chih-Hsin Hung, Tung-Nan Liao, Chien-Sen Liao

**Affiliations:** 1 Department of Medical Laboratory, Kaohsiung Municipal Siaogang Hospital, Kaohsiung 81267, Taiwan; 880475@kmuh.org.tw (Y.-P.C.); 870602@kmuh.org.tw (C.-C.L.); 890049@kmuh.org.tw (C.-M.K.); 2 Institute of Biotechnology and Chemical Engineering, I Shou University, Kaohsiung 84001, Taiwan; d91623404@ntu.edu.tw (R.C.); chhung@isu.edu.tw (C.-H.H.); 3 Department of Recreation Sports Management, Tajen University, Pingtung 90741, Taiwan; 4 Department of Emergency Medicine, Kaohsiung Veterans General Hospital, Kaohsiung 81362, Taiwan; 5 Department of Medical Laboratory Science and Biotechnology, Chung-Hwa University of Medical Technology, Tainan 71703, Taiwan; tn20030303@yahoo.com.tw; 6 Department of Civil and Ecological Engineering, I Shou University, Kaohsiung 84001, Taiwan; 7 Department of Food and Human Health Sciences, Osaka City University, Osaka 558-8585, Japan

**Keywords:** multidrug-resistant organisms, vancomycin-resistant *enterococcus*, carbapenem-resistant *acinetobacter baumannii*, methicillin-resistant *staphylococcus aureus*

## Abstract

This study evaluated the prevalence of clinical multidrug-resistant organisms (MDROs) and analyzed correlations between MDROs and patient characteristics in a regional teaching hospital of Taiwan. A retrospective comparative case-control study was conducted from January 2016 to August 2018 by collecting data from 486 hospitalized and non-hospitalized patients (M = 286, F = 200), including patient gender and age, microbial species, and antibiotic susceptibility. The results indicated that at least one MDRO was isolated from 5.3–6.3% of patients (*p* < 0.05), with an average age of 61.08 years. Of the MDROs strains, vancomycin-resistant *enterococcus* and carbapenem-resistant *acinetobacter baumannii* increased annually (*p* < 0.002 and *p* < 0.012, respectively). Three factors of age (over 60 years), treatment in an intensive care unit (ICU), and specimen category were statistically significant (*p* < 0.039, *p* < 0.001 and *p* < 0.001, respectively) and indicated that elderly patients in an ICU have a higher risk of being infected by MDROs. The outpatients infected by methicillin-resistant *staphylococcus aureus* (MRSA) were more frequent than inpatients, implying the existence of community-acquired MRSA strains. The results of this study could provide valuable information for the detection and colonization of multidrug-resistant organisms in hospital infection control systems.

## 1. Introduction

Multidrug-resistant organisms (MDROs) are pathogenic bacteria resistant to more than one kind of antibiotic [1]. Common MDROs include carbapenem-resistant *acinetobacter baumannii* (CRAB) [2], carbapenem-resistant *pseudomonas aeruginosa* (CRPA) [3], methicillin-resistant *Staphylococcus aureus* (MRSA) [4], and vancomycin-resistant *enterococcus faecium* (VREfm) [5]. In 1997, the antimicrobial surveillance program SENTRY [6] indicated that the proportion of drug-resistant bacteria in intestinal microbiota had substantially increased so that there were only a limited range of effective antibiotics and in some cases no suitable antibiotics for use in clinical treatments. MDROs will spread widely in hospitals through the selective pressure due to broad use of antibiotics among patients with low immunity or severe illnesses, and through bacterial colonization.

During the past several decades, the prevalence of MDROs in hospitals and medical centers of the world has increased steadily. According to statistics from the National Nosocomial Infection Surveillance (NNIS) system, MRSAs originating in intensive care units (ICUs) accounted for 50% and 59.5% of *S. aureus* isolates in 1999 and 2003, respectively. VREs from ICUs accounted for almost 25% of *Enterococcus* isolates in 1999, and 28.5% in 2003. Similarly, from 1999 to 2003, *P. aeruginosa* resistant to fluoroquinolone antibiotics increased from 23% to 29.5% in ICUs. A survey of 15 Brooklyn hospitals in 1999 found that 53% of *A. baumannii* strains exhibited resistance to carbapenems [7]. According to the Taiwan nosocomial infections surveillance information system monitoring report from 2008 to 2017, the CRAB, carbapenem-resistant Enterobacteriaceae (CRE), CRPA and VRE distribution of healthcare-associated infections of ICUs in medical centers and regional hospitals have increased year by year, especially VRE, whereas MRSA infections have decreased [8].

In 2013, Xu [9] suggested a close relationship (*p* < 0.001) between anti-pseudomonal carbapenems usage and the prevalence of imipenem- and meropenem-resistant *P. aeruginosa* to several classes, rather than to all antimicrobial agents in the hospital. On the other hand, Dermota et. al. [10] highlighted the importance of maintaining surveillance of community-associated MRSA (CA-MRSA) at the national level and considering CA-MRSA as a public health threat. In 2015, Wei [11] indicated that infection could lead to high mortality among neonates in a neonatal intensive care unit (NICU). Since infections are more critical for individuals with neutropenia or thrombocytopenia, in 2018, Macesic et al. [12] adopted the innovative use of active surveillance and whole-genome sequencing to represent the patterns of MDRO colonization dynamics and infection in liver transplant recipients. MDRO infection is a critical obstacle in public health management in both hospitals and the community. 

Implementing nosocomial infection surveillance is of vital importance [13,14] for controlling MDROs, since it can detect emergent pathogens, monitor long-term epidemiological prevalence and assess the effectiveness of interventions. MDRO monitoring strategies include: (1) understanding the results of regular clinical acquired microbiological experiments; (2) implementing active culturing surveillance of incoming asymptomatic patients; (3) strengthening the management of laboratory-resistant strains. Adopting these strategies in combination can reduce cross-propagation and infection rates in various areas of the hospital. It can also reduce excessive use of medical resources and permit the use of standard antibiotics to achieve patient-centered care [15]. 

In Taiwan, most research focused on the analysis of specific MDROs but not comprehensive epidemiological studies of MDROs in regional teaching hospitals. Therefore, this study investigates the prevalence of MDROs in a regional teaching hospital and explores the correlation between characteristics of patients and isolates of MDROs to discuss current MDRO epidemiology in Taiwan. These results can be an important indicator of nosocomial infection control, provide relevant data to supplement medical care-related infection data, help flag abnormal warnings for suitable interventions and generally support public health in Taiwan.

## 2. Materials and Methods

### 2.1. Bacterial Isolation

This retrospective case-control study was conducted in a regional teaching hospital with 486 patient beds that is an important emergency medical institution in Kaohsiung, Taiwan (R.O.C.). A total of 486 strains were collected from pus (213, 43.8%), urine (92, 18.9%), sputum (76, 15.6%) and other specimens (105, 21.6%) of clinical patients from January 1, 2016 to August 31, 2018. Patients who were identified as having the first infection of an MDROs strain were included and the average age of MDRO-infected patients was 61.08 ± 23.8 years. The positive rates of MDROs-infected patients from different years were analyzed by WHONET.

### 2.2. Phenotypic Antibiotic Susceptibility Testing

Antibiotic susceptibility testing of all isolates was determined by MICRONAUT susceptibility testing (AST) (Merlin, Bornheim, Germany) according to the manufacturer’s recommendations to determine the minimum inhibitory concentration (MIC) of 14 antimicrobial agents in serial dilutions of antibiotics. An overnight culture of bacteria was suspended in NaCl solution (0.9%) to obtain turbidity corresponding to 0.5 McFarland Standard (Dr. Lange, CADAS photometer 30, Berlin, Germany). The bacterial suspension was diluted to a final concentration of about 10^6^–10^7^ CFU/mL, then the diluted suspension was distributed into each well of the detection plate (100 μL) and incubated at 37 °C for 18 to 24 h. The plate was scanned with an illuminometer (Merlin) at a wavelength of 620 nm. Optical density > 0.1 was interpreted as an indication of growth. The MIC of antibiotic susceptibility was according to the expert system MCN-6 (Merlin) and CLSI (Clinical and Laboratory Standards Institute) specifications. *E. coli* ATCC 25922, *E. coli* ATCC 35218 and *K. pneumoniae* ATCC 700603 were used as positive and negative control groups.

### 2.3. Antibiotic Susceptibility and Species Identification of Bacterial Isolates

Clinical specimens were inoculated on proper or selective medium to isolate the harboring bacteria. Bacterial isolates needed to be confirmed for antibiotic susceptibility and to identify the species. VITEK® 2 Compact (bioMérieux, Inc, Hazelwood, Mo.) is a commonly used commercial kit that was adopted by clinical standard examination to determine susceptibility to the antibiotics and to identify the species of the bacterial isolates. After the bacteria were pre-treated with the VITEK kit, they were analyzed by the VITEK® 2 Compact (bioMérieux, Inc, Hazelwood, Mo.). All identification stages from reading to the recording of results are automated, thereby optimizing workflow. As the system operates with bar-coded cards, full traceability is ensured and the risk of transcription errors is minimized. VITEK® 2 Compact GN (bioMérieux, VITEK 2 AST-N339 REF419341, Marcy l’Etoile, France) was used for identifying CRE, CRAB and CRPA, and Compact GP (bioMérieux VITEK 2 AST-P627 REF414124, Marcy l’Etoile, France) was used for identifying VREfm and MRSA. Antibiotic susceptibility was based on those defined by the Clinical and Laboratory Standards Institute (CLSI) guidelines 2014 (M100-S21). The MIC of antibiotic susceptibility was determined according to the expert system MCN-6 (Merlin, Diagnostics, Bornheim-Hersel, Germany) and CLSI (Clinical and Laboratory Standards Institute, Wayne, PA, USA) specification.

### 2.4. Statistical Analysis

The variation of each MDRO strain within different years was evaluated by the chi-square test to obtain trends. Multivariate analysis was performed to evaluate the epidemiological association between MDRO strains and characteristics of infected patients. All tests were two-tailed with a significance level of *p* < 0.05. SPSS software statistical version 24 (IBM, New York, NY, USA) was used for statistical analysis.

## 3. Results 

### 3.1. Case Analysis of MDRO-Infected Patients

**Figure 1:**
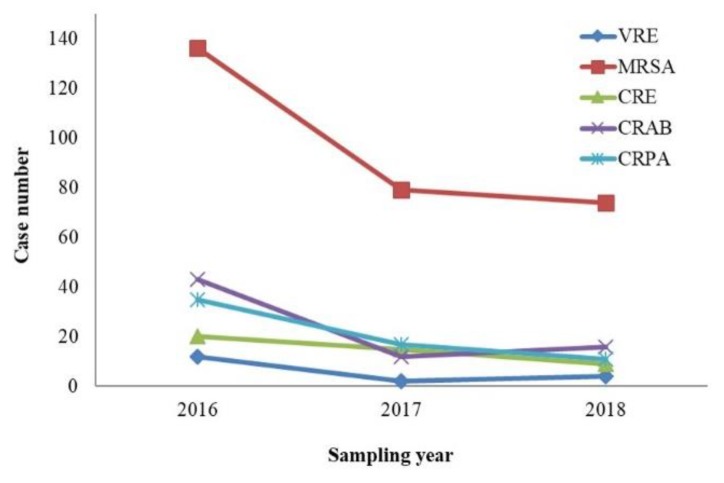
Case analysis of MDRO-infected patients from 2016 to 2018. MDRO = multidrug-resistant microorganism, VRE = vancomycin-resistant *Enterococci* spp. MRSA = methicillin-resistant *Staphylococcus aureus,* CRE = carbapenem-resistant Enterobacteriaceae, CRAB = carbapenem-resistant *acinetobacter baumannii*, CRPA = carbapenem-resistant *pseudomonas aeruginosa*.

Figure 1 shows that case numbers of MDRO-infected patients decreased from 2016 to 2018. In 2016, there was a total of 246 MDRO-infected patients with 136 MRSA infections and 43 CRAB infections. In 2017, there was a total of 125 MDRO-infected patients with 79 MRSA infections and 17 CRPA infections. From January to August 2018, there was a total of 114 MDRO-infected patients with 74 MRSA infections and 16 CRAB infections. These data show that MRSA was the major MDRO with the highest infection rate.

**Figure 2:**
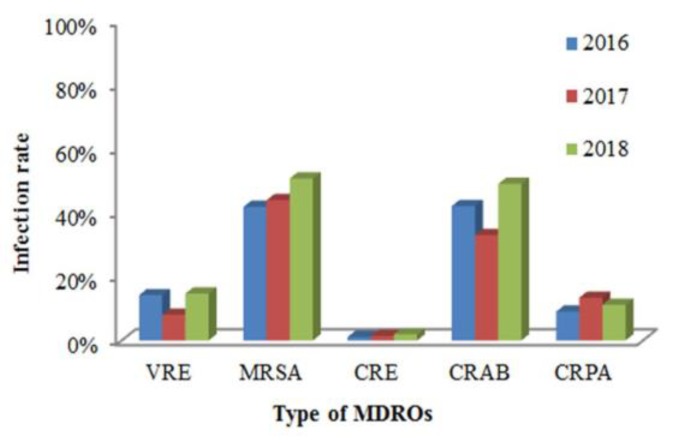
Infection rates of MDROs in Kaohsiung Municipal Siaogang Hospital from 2016 to 2018.

Figure 2 shows infection rates of MDRO-infected patients from January 2016 to August 2018. Within this period, there was a total of 486 MDRO-infected patients with 92 cases (18.9%) found in urine, 213 cases (43.8%) in pus, 76 cases (15.6%) in sputum and 105 cases (21.6%) in others. The species of isolates in 2016, 2017 and 2018 were 14.2%, 8.0% and 14.7% of VRE, 42.1%, 33.0% and 49.0% of CRAB, 41.8%, 43.9% and 50.7% of MRSA, 1.1%, 1.4% and 1.9% of CRE, 9.0%, 13.4% and 11.2% of CRPA. The chi-square test showed that VRE (*p* < 0.001) and CRAB (*p* < 0.012) were significantly increased, but there was no significant difference in MRSA (*p* = 0.821), CRE (*p* = 0.210) and CRPA (*p* = 0.830).

### 3.2. Clinical and Epidemiological Characteristics of Patients

Using multivariate analysis, the MDRO-infection risk based on gender, age, sample and ward category and station is shown on Table 1. Among the 486 MDRO-infected patients, patients infected by VER, MRSA, CRE, CRAB and CRE amounted to 18, 289, 44, 71 and 63 persons. Infection risk with significant difference (*p* < 0.05) in gender was only seen in total MDRO, but there was no significant difference in the separate analysis of each drug-resistant bacterial species. On the factor of age, MRSA infection was significantly different in the age group of 40–59 but not in the group over 60 years of age. All MDRO, VER, CRE, CRAB and CRE infections showed a significant difference over the age of 60 years. In the analysis of the sample and ward categories, MDRO and MRSA infections showed significant differences in samples of pus and sputum; CRAB and CRPA were also significantly different in pus samples. Ward and station also influenced bacterial infection. Total MDRO, VER, MRSA, CRE, CRAB and CRE infections were significantly different among the stations of ICU, RCW and CNU; MRSA, CRE, and CRPA infections were also significant different in the general ward.

### 3.3. MDRO Distribution in ICU, CNU, RCW

In the ICU, NICU and respiratory care wards, a total of 60 patients were analyzed. There were 36 males (60.0%) and 24 females (40.0%) in the gender group, eight patients (13.3%) in the age group >40 years old, 21 patients in the 40–59 age group (20.0%) and 40 patients (66.7%) >60 years old; 31 (51.7%) with sputum, 10 (16.6%) with urine and 19 others (31.7%). Of the MDROs, there were three strains (5.0%) of VRE, 27 strains (45.0%) of MRSA, five strains (8.3%) of CRE, 16 strains (26.7%) of CRAB, and nine strains (15.0%) of CRPA. MDROs had the largest number of MRSA infections in the same place in ICUs, general wards and emergency department; 16 cases of CRAB (22.53% of all CRAB) and three strains of VRE (16.67% of all VRE) were found in different ICUs. Using the Stepwise method of multivariate analysis, there were significant differences in samples of the ICU and sputum (*p* < 0.05). 

### 3.4. MDRO Distribution in Non-Hospitalized and Hospitalized Patients

This investigation ran from January 2016 to August 2018. In 2016, there were 242 MDRO-infected patients (5.3%) and 4343 patients without MDRO infections (94.7%), and in 2017, there were 122 MDRO-infected patients (5.6%) and 2059 patients without MDRO infections (94.4%). From January to August in 2018, there were 122 MDRO-infected patients (6.3%) and 1813 patients without MDRO infections (93.7%). The increase in the proportion of MDRO-infected patients between 2017 and 2018 was due to the decline in the total number of people. Analyzed by GPC (Gram-positive cocci), there were 617 inpatients and 382 outpatients with 179 (29%) and 128 (33.5%) cases of VRE and MRSA, respectively. Analyzed by GNB (Gram-negative bacteria), there were 1845 inpatients and 2135 outpatients, with 38 (2.1%) and seven (0.3%) CRE-infected cases, respectively. Analyzed by GNF (glucose-nonfermenter bacteria), there were 395 inpatients and 123 outpatients, with 111 (28.1%) and 23 (18.7%) CRAB-infected and CRPA-infected cases, respectively. These results indicated that inpatients are more infected with MDROs than MRSA.

Table 2 shows that in GPC-infected patients, the MRSA infection rate (33%) of non-hospitalized patients was higher than the rate of hospitalized patients (26.4%). This could suggest that the increased MRSA infection was community-acquired, so that MRSA prevalence is higher in the ER and OPD (without hospitalization). With other MDRO species, the hospitalized proportion was much higher than the non-hospitalized group.

## 4. Discussion

### 4.1. Resistance of MDROs Isolated from Patients with HAIs Over Time

In Southeast Asia, the cumulative incidence of healthcare-associated infections caused by *A. baumannii* is substantially higher than that reported in other regions, especially carbapenem-resistant *A. baumannii* (CRAB) (64.91%) and multidrug-resistant *A. baumannii* (MDR-AB) (58.51%). This review found a dose-response relationship between different degrees of resistant mechanisms of *A. baumannii* and the infection-caused mortality rate [16]. The isolation rates of MDROs in this hospital have increased substantially, from 5.6% in 2016 to 6.3% in 2018. Our results show that isolation rates for CRAB (*p* < 0.012) and VRE (*p* < 0.001) are increasing; however, those for CRE and CRPA are not increasing (Figure 1 and Figure 2) compared with Taiwan’s nosocomial infection surveillance information system. This difference might be due to different hospital properties, resulting in different isolation rates of MDROs. 

The results indicate that inappropriate antibiotic utilization accelerates the evolution, gene mutation or antibiotic gene transfer of microorganisms to generate drug-resistant mechanisms. This multiplies the difficulties in antibiotic selection and increases the isolation rates and epidemiology of MDROs. Now, the drug-resistance of MDROs evolves so quickly that the development of new antibiotics and the revitalization of old antibiotics cannot keep up with the mutation of drug-resistant bacteria [17]. 

In the United States, invasive MRSA infections decreased from 6.5 to 4.2 per 100 hemodialysis patients from 2005 to 2011 [18]. Among cases identified from 2009 to 2011, 70% were hospitalized in the year prior to infection, suggesting that efforts to control MRSA in hospitals might have contributed to the decline [18]. In the United Kingdom, isolation rates of MRSA have also been reduced due to enhanced hand hygiene [19]. Infection data from the US National Health Safety Network from 2006 to 2015 showed that the proportion of Enterobacteriaceae infections of CRE remained low and decreased with time, and the percentage of CRE decreased by 15% per year [19]. This suggests that early positive responses to CRE-specific infection prevention recommendations from 2009 could have slowed the emergence and even reduced the incidence of resistant pathogen infections. In 2017, our hospital began adherence to recommendations of the Joint Commission on Accreditation of Healthcare Organizations [20] to establish annual goals of patient safety that were actively coordinated with the Department of Health and Welfare by implementing hand hygiene and the concept of care bundles [21] to improve clinical care, reduce care-related infections and increase patient prognosis. These interventions might be associated with the significant decrease in MDRO incidence (Figure 1).

### 4.2. Association of Clinical and Epidemiologic Characteristics of Patients with MDRO 

This study shows a higher risk of MDRO infection for patients in ICUs, the elderly or immunocompromised patients (Table 1). These data are consistent with other research results, indicating that risk assessment for infection and the selection of antibiotic treatment for patients are essential to control the propagation of MDROs [22]. Health authorities and hospital administrators should emphasize the development of infection control programs, consider regional epidemiology and integrate all aspects. There should also be greater domestic and international cooperation providing feedback of epidemiological data to improve the understanding of MDROs and control MDRO propagation. 

### 4.3. Association of Community-Associated MRSA (CA-MRSA) and Hospital-Acquired MRSA (HA-MRSA) 

Our data show that the proportion of MRSA-infected cases in GPC (33.0%) is higher in outpatients than inpatients (26.4%) (Table 2), which is consistent with Taiwan’s nosocomial infections surveillance information system monitoring reports from 2008 to 2017. This implies an association of community-associated MRSA (CA-MRSA) and hospital-acquired MRSA (HA-MRSA), which might be a problem for both inpatients and outpatients. Previous research showed that children in the community are increasingly influenced by CA-MRSA [19]. The higher severity of CA-MRSA is due to its ability to produce toxic Panton–Valentine leukocidin (PVL), which is associated with the *staphylococcal cassette chromosome mec* (*SCCmec*) *IV* gene. Due to rapid genetic adaptation, CA-MRSA is emerging as a global public health threat. In sharp contrast to HA-MRSA, the propagation control of CA-MRSA is complex, forcing clinicians to use advanced antibiotic management skills [23,24]. Other research shows that community-related *S. aureus* strains from bloodstream infection (BSI) have considerable genetic diversity in children, identifying major genotypes of CA-MRSA and CA-MSSA (community-acquired methicillin-sensitive *S. aureus*), and finding a high prevalence of CA-MRSA [25]. Moreover, major genotypes are often associated with specific antibiotic resistance and toxin gene profiles. Understanding the molecular characteristics of these strains might give further insight into the transmission of BSI *S. aureus* in children in Chinese communities [25]. Therefore, MRSA colonization and the prevalence of MRSA infection in the surrounding community might affect strategies for the clinical management of MRSA in healthcare settings [13].

## 5. Conclusions

In summary, first, the results of this study showed that isolation rates of CRAB and VRE are on the rise. Second, the risk of MDROs infection in patients who are admitted to ICUs, elderly or immunocompromised is higher. Thirdly, the proportion of MRSA-infected cases in GPC is higher in outpatients than inpatients. Infection control continues to be involved, and strategies to actively monitor laboratory resistant strains need to be strengthened. This study describes the epidemiological distribution of MDROs in a regional teaching hospital in Taiwan, indicating the importance of monitoring the long-term prevalence of MDROs. Further experimental study design is also needed, such as the analysis of MDRO homology by pulse-field gel electrophoresis (PFGE) [26] or the evolution of MDROs by multilocus sequence typing (MLST) [27] to understand the pathogenicity mechanisms of drug-resistant genotypes. A previous study showed that different carbapenem-resistant genes of CRAB had been horizontally transferred to clonal CRAB and reduced the susceptibility of *A. baumannii* to carbapenem [28]. Therefore, clinical MRSA strains should be actively monitored and the major antibiotic-resistant genes carried by HA-MRSA and CA-MRSA should be analyzed as a reference for clinical antibiotic selection. The relationship of CA-MRSA and control strategies of MRSA in medical settings should also be further explored [29]. The results of this study could provide valuable information for the detection and colonization of multidrug-resistant organisms in hospital infection control systems.

## Figures and Tables

**Table 1:** Clinical and epidemiologic characteristics of patients whose samples were colonized with association between MDRO and covariates.

Epidemiologic Variable		Total MDRO		VRE			MRSA	CRE		CRAB		CRPA	
		Adjusted OR	95% CI	Adjusted OR	95% CI	Adjusted OR	95% CI	Adjusted OR	95% CI	Adjusted OR	95% CI	Adjusted OR	95% CI
Gender	female	ref.		ref.		ref.		ref.		ref.		ref.	
	male	1.23	1.01–1.5 *	1.31	0.48–3.55	1.10	0.85–1.42	1.84	0.98–3.47	1.62	0.95–2.75	1.00	0.58–1.70
Age (years)	<40	ref.		ref.		ref.		ref.		ref.		ref.	
	40–59	0.85	0.63–1.16	NA		0.60	0.42–0.84 *	1.88	0.61–5.75	3.13	0.59–16.4	1.49	0.39–5.69
	≥60	1.75	1.37–2.23 *	NA		0.85	0.64–1.14	4.08	1.67–9.96 *	15.8	3.78–66.00 *	7.67	2.98–19.70 *
Sample	Urine	ref.		ref.		ref.		ref.		ref.		ref.	
	Pus	9.83	7.52–12.8 *	0.34	0.07–1.57	51.90	31.3–86.00 *	1.29	0.57–2.88	2.39	1.01–5.64*	0.70	0.26–1.88
	Sputum	4.33	3.08–6.10 *	NA		8.51	4.29–16.80 *	0.65	0.23–1.83	6.77	3.33–13.70*	2.29	1.17–4.46 *
	Others	2.73	2.04–3.65 *	0.11	0.01–0.97 *	10.10	5.95–17.10 *	1.14	0.51–2.51	2.02	0.91–4.44	1.13	0.56–2.27
Station	OPD, ER	ref.		ref.		ref.		ref.		ref.		ref.	
	ICU, RCW, CNU	2.3	1.65–3.20 *	6.66	1.49–29.70 *	1.62	1.01–2.59 *	3.80	1.08–13.30 *	5.41	2.36–12.40 *	6.28	2.74–14.30 *
	General Ward	1.05	0.84–1.31	1.1	0.85–1.42	0.64	0.49–0.83 *	4.35	1.89–10.00 *	2.01	0.98–4.13	2.40	1.21–4.77 *

MDRO = multidrug-resistant microorganism. VRE = vancomycin-resistant *enterococci* spp. MRSA = methicillin-resistant *staphylococcus aureus.* CRE = carbapenem-resistant *enterobacteriaceae.* CRAB = carbapenem-resistant *a. baumannii.* CRPA = carbapenem-resistant *pseudomonas aeruginosa.* Ref. = reference.95% CI = 95% confidence interval. Adjusted OR = adjusted odds ratio. * = *p* < 0.05.

**Table 2:** MDRO distribution in non-hospitalized and hospitalized patients.

Isolates	Non-Hospitalization		Hospitalization	
	N	(%)	N	(%)
GP	(N = 382)		(N = 617)	
*Entero.* spp.	93	(24.3)	242	(39.2)
VRE	2	(0.5)	16	(2.6)
SA	161	(42.1)	196	(31.8)
MRSA	126	(33.0)	163	(26.4)
GNB	(N = 2135)		(N = 1845)	
*E. coli*	1801	(84.4)	1333	(72.2)
KP	282	(13.2)	364	(19.7)
*E. cloace*	45	(2.1)	110	(6.0)
CRE	7	(0.3)	38	(2.1)
GNF	(N = 123)		(N = 395)	
AB	15	(12.2)	51	(12.9)
CRAB	11	(8.9)	60	(15.2)
PA	85	(69.1)	233	(59.0)
CRPA	12	(9.8)	51	(12.9)

## References

[1] Boldt A.C., Schwab F., Rohde A.M., Kola A., Bui M.T., Märtin N., Kipnis M., Schröder C., Leistner R., WiesePosselt M. (2018). Admission prevalence of colonization with third-generation cephalosporin-resistant Enterobacteriaceae and subsequent infection rates in a German university hospital. PLoS ONE.

[2] Ben-Chetrit E., Wiener-Well Y., Lesho E., Kopuit P., Broyer C., Bier L., Assous M.V., Benenson S., Cohen M.J., McGann P.T. (2018). An intervention to control an ICU outbreak of carbapenem-resistant Acinetobacter baumannii: Long-term impact for the ICU and hospital. Crit. Care.

[3] Cobos-Trigueros N., Solé M., Castro P., Torres J.L., Hernández C., Rinaudo M., Fernández S., Soriano Á., Nicolás J.M., Mensa J. (2015). Acquisition of Pseudomonas aeruginosa and its resistance phenotypes in critically ill medical patients: Role of colonization pressure and antibiotic exposure. Crit. Care.

[4] Bal A.M., Gould I.M. (2005). Antibiotic resistance in Staphylococcus aureus and its relevance in therapy. Expert Opin. Pharm..

[5] Chen C.H., Lin L.C., Chang Y.J., Chang C.Y. (2017). Clinical and microbiological characteristics of vancomycin-resistant *Enterococcus faecium* bloodstream infection in Central Taiwan. Medicine.

[6] Pfaller M.A., Sader H.S., Flamm R.K., Castanheira M., Mendes R.E. (2018). Oritavancin in vitro activity against gram-positive organisms from European and United States medical centers: Results from the SENTRY Antimicrobial Surveillance Program for 2010–2014. Diagn. Microbiol. Infect. Dis..

[7] Richards M.J., Edwards J.R., Culver D.H., Gaynes R.P. (2000). Nosocomial infections in combined medical-surgical intensive care units in the United States. Infection Control & Hospital Epidemiology. Infect. Control Hosp. Epidemiol..

[8] Tseng S.H., Lee C.M., Lin T.Y., Chang S.C., Chuang Y.C., Yen M.Y., Hwang K.P., Leu H.S., Yen C.C., Chang F.Y. (2012). Combating antimicrobial resistance: Antimicrobial stewardship program in Taiwan. J. Microbiol. Immunol..

[9] Xu J., Duan X., Wu H., Zhou Q. (2013). Surveillance and correlation of antimicrobial usage and resistance of Pseudomonas aeruginosa: A hospital population-based study. PLoS ONE.

[10] Dermota U., Zdovc I., Strumbelj I., Grmek-Kosnik I., Ribic H., Rupnik M., Golob M., Zajc U., Bes M., Laurent F. (2015). Detection of methicillin-resistant Staphylococcus aureus carrying the mecC gene in human samples in Slovenia. Epidemiol. Infect..

[11] Wei H.M., Hsu Y.L., Lin H.C., Hsieh T.H., Yen T.Y., Lin H.C., Su B.H., Hwang K.P. (2015). Multidrug-resistant Acinetobacter baumannii infection among neonates in a neonatal intensive care unit at a medical center in central Taiwan. J. Microbiol. Immunol..

[12] Macesic N., Gomez-Simmonds A., Sullivan S.B., Giddins M.J., Ferguson S.A., Korakavi G., Leeds D., Park S., Shim K., Sowash M.G. (2018). Genomic Surveillance Reveals Diversity of Multidrug-Resistant Organism Colonization and Infection: A Prospective Cohort Study in Liver Transplant Recipients. Clin. Infect. Dis..

[13] Weinstein R.A. (1998). Nosocomial infection update. Emerg. Infect. Dis..

[14] Murray J., Agreiter I., Orlando L., Hutt D. (2018). BMT Settings, Infection and Infection Control. the European Blood and Marrow Transplantation Textbook for Nurses.

[15] Hara G.L., Kanj S.S., Pagani L., Abbo L., Endimiani A., Wertheim H.F.L., Amábile-Cuevas C., Tattevin P., Mehtar S., Cardoso F.L. (2016). Ten key points for the appropriate use of antibiotics in hospitalised patients: A consensus from the Antimicrobial Stewardship and Resistance Working Groups of the International Society of Chemotherapy. Int. J. Antimicrob. Agents.

[16] Ling M.L., Apisarnthanarak A., Madriaga G. (2015). The burden of healthcare-associated infections in Southeast Asia: A systematic literature review and meta-analysis. Clin. Infect. Dis..

[17] Marchaim D., Chopra T., Bogan C., Bheemreddy S., Sengstock D., Jagarlamudi R., Malani A., Lemanek L., Moshos J., Lephart P.R. (2012). The burden of multidrug-resistant organisms on tertiary hospitals posed by patients with recent stays in long-term acute care facilities. Am. J. Infect. Control.

[18] Nguyen D.B., Lessa F.C., Belflower R., Mu Y., Wise M., Nadle J., Bamberg W.M., Petit S., Ray S.M., Harrison L.H. (2013). Invasive methicillin-resistant Staphylococcus aureus infections among patients on chronic dialysis in the United States, 2005–2011. Clin. Infect. Dis..

[19] Woodworth K.R., Walters M.S., Weiner L.M., Edwards J., Brown A.C., Huang J.Y., Malik S., Slayton R.B., Paul P., Capers C. (2018). Containment of Novel Multidrug-Resistant Organisms and Resistance Mechanisms-United States, 2006–2017. MMWR-Morbid. Mortal. W..

[20] Chang A., Schyve P.M., Croteau R.J., O’leary D.S., Loeb J.M. (2005). The JCAHO patient safety event taxonomy: A standardized terminology and classification schema for near misses and adverse events. Int. J. Qual. Health Care.

[21] Rello J., Lode H., Cornaglia G., Masterton R. (2010). A European care bundle for prevention of ventilator-associated pneumonia. Intensive Care Med..

[22] Martin-Loeches I., Torres A., Rinaudo M., Terraneo S., de Rosa F., Ramirez P., Diaz E., Fernández-Barat L., Li bassi G.L., Ferrer M. (2015). Resistance patterns and outcomes in intensive care unit (ICU)-acquired pneumonia. Validation of European Centre for Disease Prevention and Control (ECDC) and the Centers for Disease Control and Prevention (CDC) classification of multidrug resistant organisms. J. Infect..

[23] Sakoulas G., Eliopoulos G.M., Fowler V.G., Moellering R.C., Novick R.P., Lucindo N., Yeaman M.R., Bayer A.S. (2005). Reduced susceptibility of Staphylococcus aureus to vancomycin and platelet microbicidal protein correlates with defective autolysis and loss of accessory gene regulator (agr) function. Antimicrob. Agents Chemother..

[24] Jarraud S., Mougel C., Thioulouse J., Lina G., Meugnier H., Forey F., Nesme X., Etienne J., Vandenesch F. (2002). Relationships between Staphylococcus aureus genetic background, virulence factors, agr groups (alleles), and human disease. Infect. Immun..

[25] Wang X., Liu Q., Zhang H., Li X., Huang W., Fu Q., Li M. (2018). Molecular Characteristics of Community-Associated Staphylococcus aureus Isolates from Pediatric Patients with Bloodstream Infections Between 2012 and 2017 in Shanghai, China. Front. Microbiol..

[26] Tenover F.C., Arbeit R.D., Goering R.V., Mickelsen P.A., Murray B.E., Persing D.H., Swaminathan B. (1995). Interpreting chromosomal DNA restriction patterns produced by pulsed-field gel electrophoresis: Criteria for bacterial strain typing. J. Clin. Microbiol..

[27] Tewolde R., Dallman T., Schaefer U., Sheppard C.L., Ashton P., Pichon B., Ellington M., Swift C., Green J., Underwood A. (2016). MOST: A modified MLST typing tool based on short read sequencing. PeerJ.

[28] Higgins P.G., Dammhayn C., Hackel M., Seifert H. (2009). Global spread of carbapenem-resistant *Acinetobacter baumannii*. J. Antimicrob. Chemother..

[29] Maree C.L., Daum R.S., Boyle-Vavra S., Matayoshi K., Miller L.G. (2007). Community-associated methicillin-resistant Staphylococcus aureus isolates and healthcare-associated infections. Emerg. Infect. Dis..

